# The quality of reporting of randomized controlled trials of HuatuoZaizao pill for stroke

**DOI:** 10.3389/fphar.2022.1106957

**Published:** 2023-01-10

**Authors:** Xiao-Jie Zhang, Yi-Jing Wang, Xiao Lu, Peng-Jie Ying, Shi-Yan Qian, Jie Liang, Guo-Qing Zheng

**Affiliations:** ^1^ Department of Neurology, The Second Affiliated Hospital and Yuying Children’s Hospital of Wenzhou Medical University, Wenzhou, China; ^2^ Department of Neurology, The First Affiliated Hospital of Zhejiang Chinese Medical University (Zhejiang Provincial Hospital of Chinese Medicine), Hangzhou, China

**Keywords:** HuatuoZaizao pill, stroke, randomized controlled trial, methodology, report quality

## Abstract

**Background:** HuatuoZaizao pill (HZP), a Chinese patent medicine, is often used in the treatment of stroke. However, there is still a lack of enough evidence to recommend the routine use of HZP for stroke. This study is aimed at evaluating the quality of reporting of randomized controlled trials (RCTs) on HZP for stroke.

**Methods:** RCTs on HuatuoZaizao pill for stroke were evaluated by using Consolidated Standards of Reporting Trials (CONSORT) guidelines and CONSORT extension criteria on reporting herbal interventions (CONSORT-CHM) guidelines. Microsoft Excel 2007 and SPSS20.0 was used for statistics analyses.

**Results:** Seventeen studies involving 1801 stroke patients were identified. CONSORT-CHM has expanded 24.3% (9/37) items in CONSORT and added a small item. The average scores of CONSORT evaluation is 14.6, while the average scores of the CONSORT-CHM evaluation is 11.6. The central items in CONSORT as eligibility criterion, sample size calculation, primary outcome, method of randomization sequence generation, allocation concealment, implementation of randomization, description of blinding, and detailed statistical methods were reported in 77%, 6%, 100%, 47%, 6%, 6%, 6%, and 94% of trials, respectively. In terms of the CONSORT-CHM, none of the articles reported in detail the dosage form, origin, formula basis and so on of HZP, and only half of studies reported the outcome indicators related to Traditional Chinese Medicine syndromes.

**Conclusion:** The overall report quality of RCT related to HZP is low. HZP still needs to report higher quality RCTs to prove its effectiveness and safety.

## Introduction

Stroke, also known as acute cerebrovascular disease, often occurs in the middle-aged and elderly. It is one of the three major diseases with the highest human mortality. The disability rate of the disease is high and the prognosis is poor, which seriously affects the quality of life of patients. With the improvement of medical level, although the mortality decreases, the disability rate remains high. Common sequelae include limb weakness and speech communication disorder. The key to the treatment of ischemic stroke is to dredge the occluded blood vessels in time in the early stage and restore the blood supply to the brain, so as to save the brain cell in the ischemic penumbra. At present, stroke patients are mainly treated with western medicine. Thrombolysis is a proven effective treatmen, but with a limited time window (Yee et al., 2017). Mechanical thrombectomy, despite its wider time window, puts patients at certain complications, such as bleeding transformation, vasospasm, arterial dissection and so on (Huo and Gao, 2018). In order to improve the treatment effect of stroke, many experts and scholars try to treat stroke with traditional Chinese medicine (TCM), which have achieved satisfactory outcomes.

TCM has a long history in the treatment of stroke. HuatuoZaizao Pill (HZP) is a concentrated water honey pill made of Chuan Qiu, Wu Rongyu, borneol and other medicinal flavors. And Part I of the Chinese Pharmacopoeia 2020 edition recorded that HZP can be used to promote rehabilitation after stroke (Chinese Pharmacopoeia Commission, 2020). HZP treatment could promote functional recovery by enhancing the expression of brain-derived neurotrophic factor (BDNF) and increasing the level of neurogenesis in cerebral ischemia-reperfusion (I/R) animal (Chen, 2010a; Zheng et al., 2014; Duan et al., 2017). In addition, HZP inhibited platelet aggregation and thrombosis through *in vitro* and *in vivo* thrombus tests. Therefore, HZP has the characteristics of improving microcirculation disorder and preventing cerebral thrombosis (Liu et al., 2000). Liu and colleagues found that HZP reduced neurological deficit score and infarct volume in a rat model of cerebral ischemia-reperfusion. HZP has a protective effect on neurons and glial cells in penumbra (Liu et al., 2004). Two meta-analyses have shown that HZP can effectively restore impaired function in patients with ischemic stroke (Cai et al., 2007; Xia et al., 2012). The existing evidence may support the use of HZP in the treatment of acute ischemic stroke, but there is still insufficient evidence to recommend the routine use of HZP in the treatment of stroke.

High quality randomized controlled trial (RCTs), especially double-blind placebo-controlled trials, are generally considered to be the highest level of evidence to judge the therapeutic effect and safety of interventions. The credibility of evidence supporting treatment depends on the quality of RCTs. However, a large amount of evidence shows that the quality of RCT reports is still sub-optimal (Turner et al., 2012). So far, two meta-analyses have proved the effectiveness of HZP in the treatment of stroke. However, no study has evaluated the quality of RCTs of HZP for stroke. The report of clinical trial needs to be clear, complete and transparent. CONSORT statement (Chan and Altman, 2005) consists of a list of basic items necessary for reporting RCT and a flow chart describing the flow of subjects in the whole trial process. CONSORT Extension for Chinese Herbal Medicine Formulas 2017(CONSORT-CHM Formulas) is the addition of TCM syndrome and items according to the characteristics of TCM compound on the basis of consort 2010 statement (Pratoomsoot et al., 2015). Therefore, the purpose of this study is to evaluate the quality of the report of the RCTs of HZP in the treatment of stroke according to the CONSORT statement and the CONSORT-CHM statement.

## Methods

### Information sources and search

From the beginning to April 2022, six English and Chinese databases were searched electronically. They are Cochrane Controlled Trials Register, PubMed, EMBASE, China National Knowledge Infrastructure (CNKI), VIP Journals Database, Wanfang Database and Chinese Biomedical Database (CBM). The search queries were listed as follows: “HuatuoZaizao pill AND (stroke OR apoplexy OR cerebrovascular accident OR cerebrovascular attack OR cerebral infarction OR cerebral vascular disease)”. The Chinese database also uses the corresponding Chinese search words mentioned above for retrieval.

### Eligibility criteria

All RCTs on HZP as monotherapy or adjuvant treatment for acute, recovery and sequelae of ischemic stroke compared with at least one control group as no treatment, sham operation/placebo or routine treatment, regardless of publication status or language, were selected. The diagnosis of stroke conforms to the diagnostic criteria of the World Health Organization (Hatano, 1976). The diagnosis of stroke was verified by CT and/or MRI.

### Exclusion criteria

Exclusion criteria included animal experiments, case reports, reviews, retrospective and historical controlled studies, repeated publications, quasi-randomized trials and studies involving patients with intracerebral hemorrhage. Search is limited to English and Chinese.

### Data extraction

Two researchers were trained to study every item and multiple subitems listed in CONSORT2010 and CONSORT-CHM 2017 to ensure the correct understanding of each standard. Each report was reviewed by two independent investigators. They extracted information according to CONSORT2010 and CONSORT-CHM 2017 checklists. “1”or “0” was scored by the two authors independently to represent whether the RCT had reported the relevant item/subitem or not. “0” indicates no description of the corresponding item/subitem and “1” indicates that the author had mentioned the description of the item/subitem in the report. Investigators resolved discrepancies by consensus or consultations during the data-extraction process.

### Data analyses

We use Microsoft Excel 2007 for descriptive statistical analysis and counted the total number of RCTs corresponding to each project. The subsequent results were expressed as percentages and 95% confidence intervals (CIs) were calculated for each overall ratio. SPSS (version 20.0) is used for statistical calculation. The significance level was presumed as *p* < .05.

## Results

### Study selection

**FIGURE 1:**
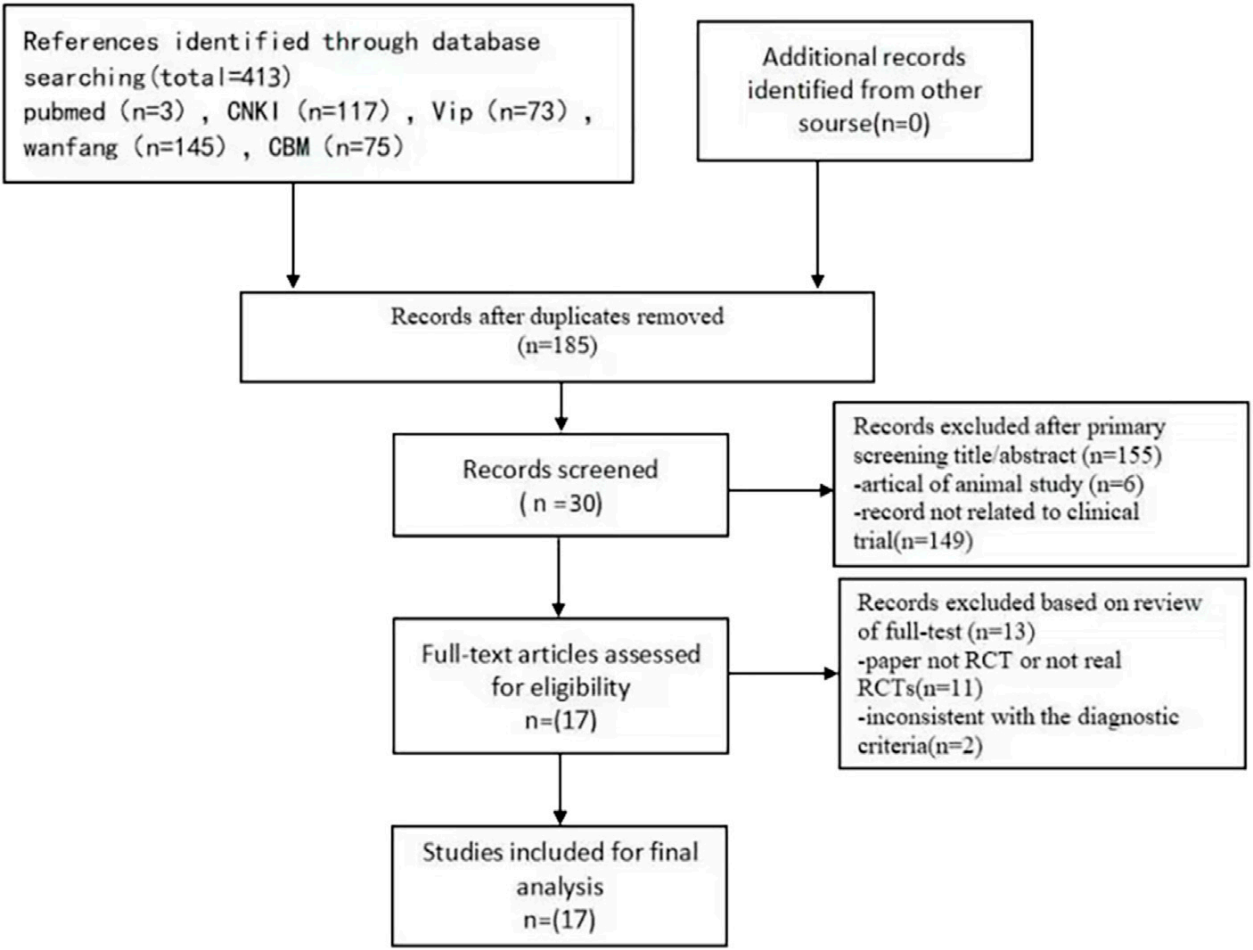
Flow diagram for the selection of articles for inclusion in the study.

A total of 413 prospective relevant articles were identified. By inspecting titles and abstracts, 383 papers were excluded for at least one of following reasons: 1) duplicate publication, 2) animal study, 3) not clinical trial. After examining the remaining literatures by reading the full text, we removed 13 papers. Of which, 11 were non-RCTs, and two were using ambiguous diagnostic criteria. Eventually, 17 eligible RCT studies were selected for the final analysis (Figure 1).

### Study characteristics

Seventeen studies involving 1801 stroke patients were identified. For the 1801 patients, there were 1,040 males and 761 females, with an age range from 33 to 81 years old. Sample sizes ranged from 44 to 300 participants. 17 studies were all published in Chinese. Four studies were online Master’s thesis and not formally published. The duration of treatment varied from 20 days to 1 year. Nine studies reported adverse effects. Key data are summarized in Table 1.

**TABLE 1:** The characteristics of the included 17 studies.

Included trails	Publication year	Publication language	Study designs	Sample size calculation	No.of participants (male/female); age (y)		Course of disease	Interventions (n) drug/dosage		Course of treatment
					Trial	Control		Trial	Control	
Lv et al. (2019)	2019	Chinese	RCT	No	40 (25/15); 65.0 ± 8.8	39 (22/17); 65.1 ± 8.3	<6 h	HZP + WCTs#	WCTs*(general supportive care, Antiplatelet agents, anticoagulants)	12 w
Ma (2012)	2012	Chinese	RCT	No	60; 60.6 ± 5.1	60; 60.6 ± 5.1	<7 d	HZP + WCTs#	WCTs*(general supportive care, Antiplatelet agents)	20 d
Yang and Ning (2016a)	2016	Chinese	RCT	No	43; 66 ± 12	42; 66 ± 12	<2 d	HZP + WCTs#	WCTs*(general supportive care, specialized care)	1 m
Yang and Kan (2017)	2017	Chinese	RCT	No	59 (34/25); 66.7 ± 2.7	59 (30/29); 65.2 ± 2.3	<65 d	HZP + WCTs#	WCTs*(Antiplatelet agents aspirin 0.1 g po qd)	3 m
Peng et al. (2020)	2020	Chinese	RCT	No	30 (20/10); 68.2 ± 11	30 (16/14); 69.7 ± 10	<3 m	HZP + WCTs#	WCTs*(stroke rehabilitation, cluster needling of scalp point therapy and vibration treatment)	1 m
Wang (2012)	2012	Chinese	RCT	No	50; 55.5 ± 10.4	50; 55.5 ± 10.4	<1 m	HZP + WCTs#	WCTs*(general supportive care, Antiplatelet agents)	24 w
Kan and Wu (2014)	2014	Chinese	RCT	No	57 (30/27); 65.2 ± 2.4	60 (32/67); 67.3 ± 3.2	<14 d	HZP + WCTs#	Aspirin + WCTs*(general supportive care, stroke rehabilitation)	1 y
Du and Zhang (2003)	2003	Chinese	RCT	No	102 (63/39); Mean 60	64 (37/27); Mean 60	<2 y	HZP + WCTs#	WCTs*(general supportive care, specialized care)+Renshen zaizao	1 m
Chen J (unpublished Master’s thesis) (Chen, 2010b)	2010	Chinese	RCT	No	30 (17/13); 58.7 ± 8.0	30 (16/14); 57.9 ± 8.5	<1 m	HZP + WCTs#	WCTs*(general supportive care, Antiplatelet agents)	6 m
Su L (unpublished Master’s thesis) (Su, 2011)	2011	Chinese	RCT	No	100 (56/44); 60.8 ± 8.0	94 (55/39); 59.9 ± 9.0	<1 m	HZP + WCTs#	WCTs*(general supportive care, Antiplatelet agents aspirin 0.1 g po qn, stroke rehabilitation)	24 w
Zhang (2018)	2018	Chinese	RCT	No	42 (24/18); 62 ± 1.58	42 (22/20); 61 ± 1.62	<6 m	HZP + WCTs#	WCTs*(general supportive care, specialized care)	2 m-3 m
HuS (unpublished Master’s thesis) (Hu, 2019)	2019	Chinese	RCT	No	31 (18/13); 66.5 ± 7.0	33 (20/11); 65 ± 7.4	<20 m	HZP + WCTs#	WCTs*(specialized care, Antiplatelet agents aspirin 0.1 g po qd)	1 m
Yang and Ning (2016b)	2016	Chinese	RCT	No	40; 68 ± 12	39; 68 ± 14	<72 h	HZP + WCTs#	WCTs*(general supportive care, specialized care)	1 m
Bai Y (unpublished Master’s thesis) (Bai, 2011)	2011	Chinese	RCT	Yes	30 (20/10); Mean 62.0	30 (18/12); Mean 59.3	<1 m	HZP + WCTs#	WCTs*(general supportive care, Antiplatelet agents aspirin 0.1 g po qn)	6 m
Jing (2013)	2007	Chinese	RCT	No	23 (13/10); 47.5 ± 8.2	23 (12/11); 45.3 ± 6.3	<3 d	HZP + WCTs#	WCTs*(Ozone-AHT, general supportive care, specialized care)	40 d
Xie and He (2019)	2019	Chinese	RCT	No	100 (63/37); 66.8 ± 2.9	100 (64/36); 66.8 ± 2.9	<19 d	HZP + WCTs#	WCTs*(general supportive care, specialized care)	40 d
Wang et al. (2022)	2022	Chinese	RCT	No	26 (19/7); 58.50 ± 8.19	18 (8/10) 60.56 ± 7.27	<6 m	HZP + WCTs#	WCTs*(general supportive care, Antiplatelet agents)	3 m

D, day; m, month; RCT, randomizedcontrolledtrial; w, week; WCTs, western conventional treatments; y, year. #: the same as the control group; WCT* refer to the combination of needed therapies of the following aspects: 1) General supportive care mainly include: A. airway, ventilatory support and supplemental oxygen, B. cardiac monitoring and treatment, C. temperature, D. blood pressure, E. blood sugar and F. nutrition; 2) Specialized care mainly include a variety of measures to improve cerebral blood circulation (such as antiplatelet agents, anticoagulants, fibrinogen-depleting agents, volume expansion and vasodilators, except thrombolytic agents) and neuroprotective agents; 3) Treatment of acute complications mainly include: A. brain edema and elevated intracranial pressure, B. seizures, C. dysphagia, D. pneumonia, E. voiding dysfunction and urinary tract infections and F. deep vein thrombosis.4) Stroke rehabilitationfibrinogen-depleting agents, volume expansion and vasodilators, except thrombolytic agents) and neuroprotective agents; 3) Treatment of acute complications mainly include: A. brain edema and elevated intracranial pressure, B. seizures, C. dysphagia, D. pneumonia, E. voiding dysfunction and urinary tract infections and F. deep vein thrombosis.4) Stroke rehabilitation.

**FIGURE 2:**
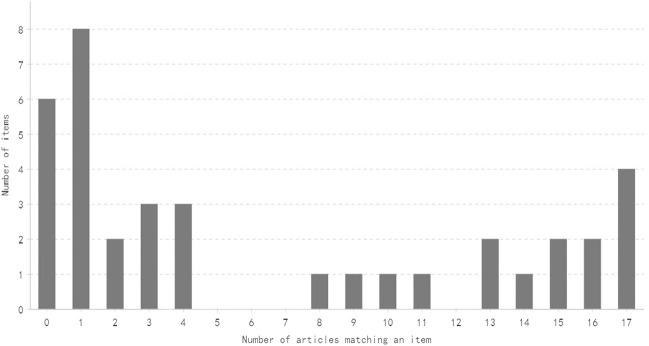
Distribution of the number of CONSORT items satisfied by a specific number of articles.

**FIGURE 3:**
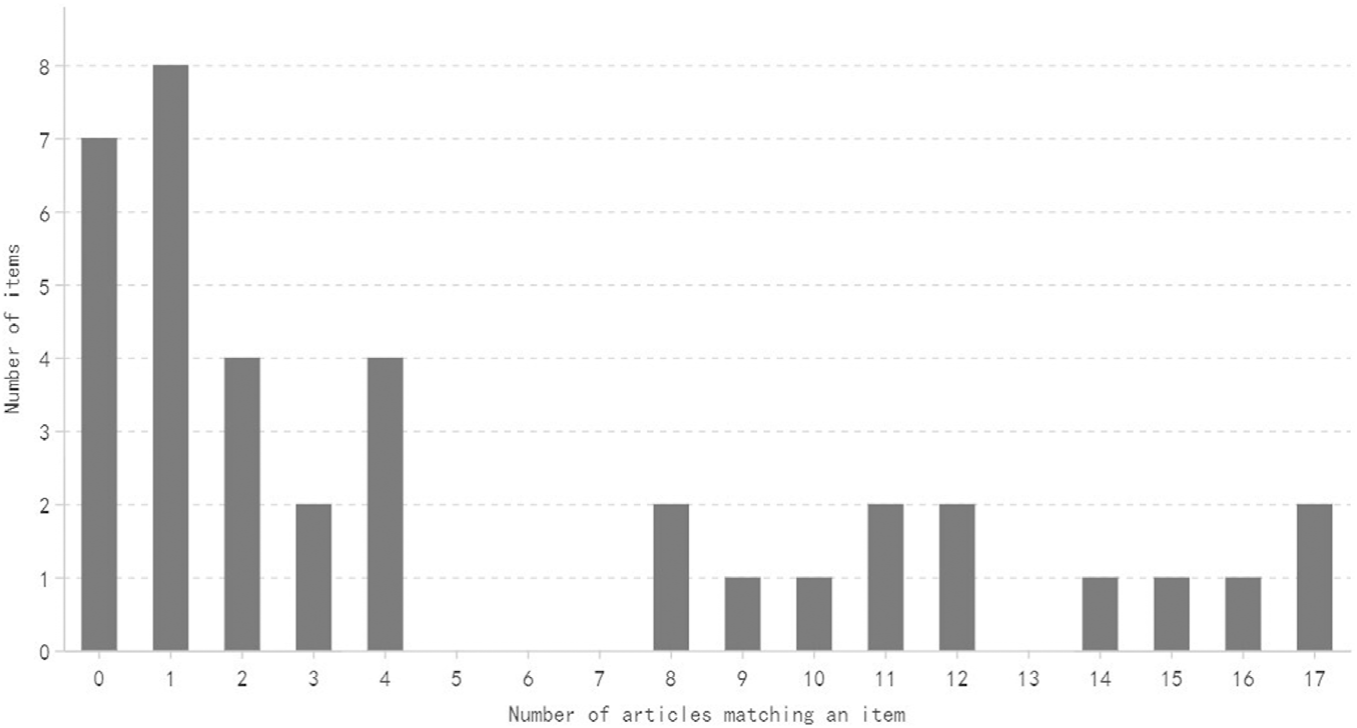
Distribution of the number of CONSORT-CHM items satisfied by a specific mumber of articles.

The distribution of the number of items satisfied by a specific number of articles is shown in Figures 2, 3. Generally speaking, most of the items are satisfied with a few articles, and this is more obvious for CONSORT-CHM.

Items reported according to CONSORT statement.

The items reported from the 17 RCTs according to CONSORT statement are summarized in Table 2.

**TABLE 2:** The reporting number and percentage for each item of the CONSORT and CONSRT-CHM checklist of the included 17 studies.

Section/Topic	Item no.	Checklist item	*n*	% (*n*/17)	95%CI
Title and abstract	1a	Identification as a randomized trial in the title	3	18	[0 to 38]
	1a*	Statement of whether the trial targets a TCM Pattern, a Western medicine–defined disease, or a Western medicine–defined disease with a specific TCM Pattern	2	12	[0 to 29]
	1b	Structured summary of trial design, methods,results, and conclusions (for specific guidance see CONSORT for abstracts)	16	94	[82 to 100]
	1b*	Illustration of the name and form of the formula used, and the TCM Pattern applied, if applicable	4	24	[1 to 46]
	1c*	Determination of appropriate keywords, including “Chinese herbal medicine formula” and “RCT”	2	12	[0 to 29]
Introduction					
Background and objectives	2a	Scientific background and explanation of rationale	13	77	[54 to 99]
	2a*	Statement with biomedical science approaches and/or TCM approaches	11	65	[39 to 90]
	2b	Specific objectives or hypotheses	11	65	[39 to 90]
	2b*	Statement of whether the formula targets a Western medicine–defined disease, a TCM Pattern, or a Western medicine-defined disease with a specific TCM Pattern	11	65	[39 to 90]
Methods					
Trial design	3a	Description of trial design (such as parallel, factorial)including allocation ratio	17	100	[100 to 100]
	3b	Important changes to methods after trial commencement (such as eligibility criteria), with reasons	0	0	[0 to 0]
Participants	4a	Eligibility criteria for participants	13	77	[54 to 99]
	4a*	Statement of whether participants with a specific TCM Pattern were recruited	12	71	[46 to 95]
	4b	Settings and locations where the data were collected	17	100	[100 to 100]
Interventions	5	The interventions for each group with sufficient details to allow replication, including how and when they were actually administered	17	100	[100 to 100]
	5*	Description(s) for different types of formulas should include specific contents	0	0	[0 to 0]
Outcomes	6a	Completely defined pre-specified primary and secondary outcome measures, including how and when they were assessed	17	100	[100 to 100]
	6a*	Illustration of outcome measures with Pattern in detail	8	47	[21 to 74]
	6b	Any changes to trial outcomes after the trial commenced, with reasons	0	0	[0 to 0]
Sample size	7a	How sample size was determined	1	6	[0 to 18]
	7b	When applicable, explanation of any interim analyses and stopping guidelines	2	12	[0 to 29]
Randomization					
Sequence generation	8a	Method used to generate the random allocation sequence	8	47	[21 to 74]
	8b	Type of randomization; details of any restriction (such as blocking and block size)	3	18	[0 to 38]
Allocation concealment mechanism	9	Mechanism used to implement the random allocation sequence (such as sequentially numbered containers), describing any steps taken to conceal the sequence until interventions were assigned	1	6	[0 to 18]
Implementation	10	Who generated the random allocation sequence, who enrolled participants, and who assigned participants to interventions	1	6	[0 to 18]
Blinding	11a	If done, who was blinded after assignment to interventions (for example, participants, care providers, those assessing outcomes)and how	1	6	[0 to 18]
	11b	If relevant, description of the similarity of interventions	2	12	[0 to 29]
Statistical methods	12a	Statistical methods used to compare groups for primary and secondary outcomes	16	94	[82 to 100]
	12b	Methods for additional analyses, such as subgroup analyses and adjusted analyses	1	6	[0 to 18]
Results					
Participant flow (a diagram is strongly recommended)	13a	For each group, the numbers of participants who were randomly assigned, received intended treatment, and were analyzed for the primary outcome	14	82	[62 to 100]
	13b	For each group, losses and exclusions after randomization, together with reasons	4	24	[1 to 46]
Recruitment	14a	Dates defining the periods of recruitment and follow-up	15	88	[71 to 100]
	14b	Why the trial ended or was stopped	3	18	[0 to 38]
Baseline data	15	A table showing baseline demographic and clinical characteristics for each group	4	24	[1 to 46]
Baseline data	16	For each group, number of participants (denominator)included in each analysis and whether the analysis was by original assigned groups	10	59	[33 to 85]
Outcomes and estimation	17a	For each primary and secondary outcome, results for each group, and the estimated effect size and its precision (such as 95% confidence interval)	0	0	[0 to 0]
	17 b	For binary outcomes, presentation of both absolute and relative effect sizes is recommended	0	0	[0 to 0]
Ancillary analyses	18	Results of any other analyses performed, including and analyses, distinguishing pre-specified from exploratory	1	6	[0 to 18]
Harms	19	All important harms or unintended effects in each group (for specific guidance see CONSORT for harms)	9	53	[27 to 79]
Discussion					
Limitations	20	Trial limitations, addressing sources of potential bias, imprecision,and, if relevant, multiplicity of analyses	4	24	[1 to 46]
Generalizability	21	Generalizability (external validity, applicability) of the trial findings	1	6	[0 to 18]
	21*	Discussion of how the formula works on different TCM Patterns or diseases	1	6	[0 to 18]
Interpretation	22	Interpretation consistent with results, balancing benefits and harms, and considering other relevant evidence	15	88	[71 to 100]
	22*	Interpretation with TCM theory	12	71	[46 to 95]
Otherinformation					
Registration	23	Registration number and name of trial registry	0	0	[0 to 0]
Protocol	24	Where the full trial protocol can be accessed, if available	1	6	[0 to 18]
Funding	25	Sources of funding and other support (such as supply of drugs), role of funders	0	0	[0 to 0]
Total mean score of CONSORT_a_			14.6 ± 4.37		
Total mean score of CONSORT-CHM_a_			11.6 ± 4.66		

CONSORT-CHM = CONSORT, extension for chinese herbal medicine formulas.

Items with * indicate that this item is CONSORT-CHM.

_a_Mean ± SD.

### Title and abstract

#### CONSORT

Only three (18%) trials can be identified as RCT after reading the title (1a). Sixteen (94%) articles had abstracts that show the structured summary of experimental design, methods, results and conclusions (1b).

#### CONSORT-CHM

Two articles (12%) stated by title that the trial targets the specific disease (1a*). Only four (24%) trials described the name, dosage form and TCM pattern applied of the compound (1b*). Only two (12%) article determined appropriate keywords, including “Chinese herbal medicine formula” and “RCT” (1c*).

### Introduction

#### CONSORT

In the included articles, 13 (77%) studies had show the scientific background and explanation of rationale (2a). And there were 11 (65%) articles that provided specific objectives or hypotheses (2b).

#### CONSORT-CHM

Eleven (65%) articles had statement with TCM approaches (2a*), and the same number (65%) of articles had statement of whether the formula targets a specific disease (2b*).

### Methods

#### CONSORT

There were two items that had not been described in any article (0%), and they were the description of significant changes in the experimental method (3b) and whether there are changes in the trial outcomes after the commencing of the experiment (6b). Four CONSORT items were described in all the included articles (100%), and they were description of trial design including allocation ratio (3a), settings and locationswhere the data was collected (4b), the interventions for each group with sufficient details to allow replication (5) and detailed description of outcome measures (6a). The proportion on the eligibility criteria for participants was 77% (4a). One (6%) article illustrate how sample size was determined (7a) and two (12%) studies provided the explanation of any interim analysis and stopping guidelines (7b), respectively.

#### CONSORT-CHM

Twelve (71%) trials stated whether participants with a specific TCM pattern were recruited (4a*). None of the articles (0%) described the Chinese herbal medicine formula in detail (5*). Eight (47%) articles reported the outcome measures related to TCM syndrome in detail (6a*). Other items in CONSORT-CHM are consistent with CONSORT.

### Randomization

#### CONSORT

Four items were only described in one article (6%). They were mechanisms used to implement the random allocation sequence (9), the detailed implementation (10), the description of blinding (11a), and methods for additional analyses (12b). Eight (47%) studies provided the method used to generate the random allocation sequence (8a). However, the proportion of the description on the type of randomization (8b) was 18%. Only 2 (12%) articles mentioned the similarity of interventions (11b). Simultaneously, sixteen (94%) papers described the detailed statistical methods (12a).

#### CONSORT-CHM

All items in this content are the same as CONSORT.

### Results

#### CONSORT

82% studies described the treatment progress with a diagram (13a) was. Four (24%) trials mentioned the losses and exclusions after randomization with explanations (13b). A total of 15 studies (88%) described the dates defining the periods of recruitment and the follow-up duration (14a). Only 3 (18%) articles illustrated the reasons of why the trial ended or was stopped (14b).

Four (24%) articles provided the description of baseline data that included underlying disease or basic demographic or clinical characteristics (15). Ten (59%) studies stated the statistics methods, including the use of intention-to-treat analysis (16). None of the articles (0%) provided the estimated effect size (17a) and absolute or relative effect sizes (17b). Only one (6%) studies offered results of any other analyses performed (18). Nine articles (53%) illustrated all important harms or unintended effects in each group (19).

#### CONSORT-CHM

All items in this content are the same as CONSORT.

### Discussion

#### CONSORT

Only four (24%) articles reported the limitation of trials (20) and one (6%) article illustrated the generalizability of the trial findings (21). However, fifteen (88%) studies offered the interpretation consistent with result (22).

#### CONSORT-CHM

Item 20 has no extension. One (6%) article provided the discussion of how the formula works on different TCM patterns on disease (21*). And twelve (71%) studies additionally offered the interpretation with TCM theory (22*).

### Other information

#### CONSORT

None of trials (0%) provided registration (23) and sources of funding (25). And only one (6%) articles illustrated where the full trial protocol can be accessed (24).

#### CONSORT-CHM

All items in this content were the same as CONSORT.

### Total mean scores

Among the 17 articles in this study, the average scores of CONSORT evaluation is 14.6, accounting for 39% of the total items, while the average score of CONSORT-CHM evaluation is 11.6, accounting for only 31% of the total items.

## Discussion

The CONSORT statement is an evidence-based,minimum set of recommendations for standardizing the results of RCT and reducing the bias of RCT research. It standardizes the publication of RCT results and improves the quality of research papers to a great extent (Moher et al., 2010). At present, more than 400 academic journals around the world have adopted the CONSORT statement, which can be used as an important reference for judging whether the article is written in a standardized manner and whether it can be officially published in the process of paper review (Moher et al., 2001; Chan and Altman, 2005; Plint et al., 2006). However, since the Research Report of the first RCT of TCM was published in 1982 (Chen et al., 1982), the quality of tens of thousands of clinical trial reports related to TCM prescriptions is not very ideal. It not only reduces the value of TCM, affects the judgment of commentators and readers on its efficacy and safety, but also causes doubts and criticism of TCM from all walks of life, and finally hinders the application and development of clinical practice and patient care (Wang et al., 2007; Wu et al., 2009; He et al., 2011; Wieland et al., 2013). Therefore, in order to improve the overall reporting quality of clinical trials of TCM,CONSORT-CHM (CONSORT extension for Chinese herbal medicine formulas 2017) has been formulated (Cheng et al., 2017). On the basis of CONSORT 2010 statement, it adds TCM syndrome and items according to the characteristics of TCM compound, adds one sub item, and expands the contents of seven items, so as to improve the reporting quality of clinical RCTs of TCM compound. CONSORT-CHM has not been widely applicated since its recent publication (Pratoomsoot et al., 2015). For studies that have adhered to the CONSORT-CHM principle, there is great improvement in transparency regarding the reporting of herbal interventions (Ornelas et al., 2018).

In this study, a few articles obtained good scores. One article obtained a high score of 26 points in CONSORT and CONSORT-CHM evaluation, but the scores of most studies were low. Only four articles and two articles in CONSORT and CONSORT-CHM reached 20 points, respectively, while the proportion of articles with 10 points and below in CONSORT and CONSORT-CHM was as high as 29.4% and 64.7%, respectively, The average CONSORT score and CONSORT-CHM score of all articles were only 14.6 and 11.6. On the whole, these articles mainly have the following deficiencies.

From the perspective of CONSORT:1. The title indicates that the corresponding article is a RCT, which can make it more easily identified. However, only three articles in this study can be seen as a RCT through the title; 2. Ideally, any study should evaluate the entire study population, but this rarely happens due to financial reasons and time constraints, and sampling is the most commonly used method. However, the study found that a large number of surveys used unrepresentative samples and incorrectly tried to extrapolate their results to the research population (Sandhu et al., 2015). Therefore, in order to achieve the external validity of the results, many methods are used to ensure that the samples studied are representative, including sample size calculation (Peduzzi et al., 2002). Relevant studies have found that if the pre-test sample size is not estimated, there is a lack of statistical ability to ensure the proper estimation of the treatment effect (Schulz and Grimed, 2005). Hence, the CONSORT team recommends reporting the details of sample size determination to determine the main results and as a sign of an appropriate test plan (Altman et al., 2002). Nevertheless, only one article in this study explains how to calculate the sample size. In this case, an effort should be made to improve the transparency of sample size calculation to improve the external effectiveness of the RCT. If the sample size calculation report has little correlation in the randomized controlled trial, it may be necessary to give up, as suggested by Bachetti (Bacchetti, 2002); 3. Relevant studies have found that sufficient randomization is a necessary measure to ensure the authenticity of the results, and blind rule is an important protective measure to reduce errors (Berger and Bears, 2003; Jelena et al., 2012). Inadequate or inaccurate allocation concealment will exaggerate the clinical impact by 41% and 30%, respectively, while the absence of a blind rule will exaggerate the treatment effect by an average of 17% (Schulz et al., 1995). And in this study, most articles lack a description of the randomization process, and the implementation of the blind method is not good. Therefore, we hope that more researchers will strengthen the implementation of randomization and blinding; 4. In the content of the results, the baseline situation of the subjects can reflect the comparability between the experimental group and the control group, but only 24% of the articles in this study explained the baseline situation. In addition, the estimated effect size or absolute and relative effect size can help readers better understand the benefits of drugs, but none of the articles in this study provide the corresponding content, so we suggest that future researchers can provide it; 5. In the discussion, only a few articles explain the limitations and extrapolation. 6. Among other things, the International Committee of Medical Journal Editors (ICMJE) requires all clinical trials must be registered to improve transparency and accountability (DeAngelis et al., 2005). However, none of the trials in this study were registered. At the same time, these articles did not explain the source of funds and conflicts of interest. Only one study provided a plan. This has affected the credibility of these articles. In addition, these articles did not indicate whether the trial was reviewed by the relevant ethics committee. They also do not explain how to select researchers. Thus we hope that future research can improve these projects, so as to ensure that the rights of subjects are protected and the credibility of the research is guaranteed.

From the perspective of CONSORT-CHM: 1. Only two articles added “HuatuoZaizao pill” and “randomized clinical trial” to the keywords. 2. None of the articles reported in detail the dosage form, origin, formula basis, etc., of HZP. 3. All articles described the outcome indicators in detail, but only 47% of them reported the outcome indicators related to TCM syndromes. 4. There are still a few articles that are not explained by the relevant theories of TCM in the discussion part.

This study still has some limitations. First, the number of trials included in this study is not enough, and there may be a risk of bias. Secondly, this study only searched the literature in Chinese and English, and finally only included the literature in Chinese, and articles in other languages may be omitted. Finally, we extract the data according to the published paper itself. This approach means that we cannot capture some preliminary tests with good quality in the test method, but are not reported in the final publication. Therefore, when evaluating the trial quality of such studies, it is necessary to review the study protocol and contact the experimenter for more information.

## Conclusion

Our research shows that the overall report quality of RCT related to HZP is low, especially in terms of title, sample size calculation, randomization and blind method, limitations, extrapolation, accessibility, trial registration and conflict of interest. The quality of reports related to TCM compound is even worse, especially the details of TCM compound and the indicators related to TCM syndrome need to be improved. Therefore, we believe that HZP still needs to report higher quality RCTs to prove its effectiveness and safety.

## Data Availability

The original contributions presented in the study are included in the article/Supplementary Material, further inquiries can be directed to the corresponding author.
